# 

**Published:** 1914-01-24

**Authors:** 


### CONTENTS.

PAGE

**The Decline in the Tuberculosis Death-rate** ... 437

**Reforms in County Council Mental Hospitals** 438

**Hospital and Institutional News** ... ... ... 439
New Clinical Laboratories at Wolverhampton—The Medical Staff of the Leicester Poor-Law Infirmary—The Proper Salary for an Infirmary Superintendent—The Payment of Hospital Chaplains—The Control of Radium in America—Early Medical Literature—The Central Poor-Law Conference, 1914—The Use of Radium in Australia—Lister Memorial Tablet at King's College—A Rate-Supported House Surgeon—A Great Private Mental Hospital—Sunday and Saturday Fund Decreases—Hospital Clerk Burnt to Death—Remarkable Gift to Ashton Infirmary—Higher Salaries for London Infirmary Staffs—Marriage and the Wassermann Reaction—Hospitals and the Workmen's Compensation Act—Problems of Discipline at Winsley Sanatorium—A Prize for Medico-Legal Work—The London Hospital's Appeal—North Staffordshire Infirmary Problems—Barbados General Hospital—Birthdays—The Insurance Act and Proprietary Medicines—This Week's Drug Market.

PAGE

**Medico-Psychology** ... ... ... ... ... 445

The Trend of Research in Mental Hospitals.

**Dental Treatment at Tuberculosis Dispensaries** 446
By a Tuberculosis Dispensary Officer.

**The Orthopædic Department** ... ... ... 447

V. The Modern Orthopædic Unit (*continued*).

**Hospitals and Vaccine Therapy** ... ... ... 449

**The Administration of the Poor-Law** ... ... 450

The Local Government Board's Annual Report.

[*See over.*]

### CONTENTS—(*continued*).

| | PAGE |
|---|---|
| **St. George's Hospital** ... ... ... ... | 451 |
| A Settlement in Sight. | |
| **Hospital Architecture and Construction** ... | 452 |
| The Alterations at Chichester Infirmary. | |
| **Reports on Hospitals of the United Kingdom—Series III.** ... ... ... ... ... ... ... | 453 |
| By Sir HENRY BURDETT, K.C.B., K.C.V.O. | |
| Huntingdon County Hospital. | |
| Bury St. Edmund's Hospital. | |
| **Raise Your Standard of Efficiency** ... ... | 454 |
| **The Editor's Letter-Box** ... ... ... ... | 455 |
| Article 10 of the Poor-Law Orders. | |
| Hospitals and the Truck Act. | |
| Inexpensive Mental Homes for the Middle Classes. | |
| A Point for Sanatoria Committees. | |
| **National Insurance Act Developments** ... ... | 457 |
| On Going Out of Insurance. | |
| **A Winter in the Upper Engadine, 1885** ... | 459 |
| By One of the First Consumptives to Visit the High Alps. | |
| **Round the World** ... ... ... ... ... | 461 |
| Fellow-workers and their Doings. | |
| **The Profession of Medicine** ... ... ... | 462 |
| Once more the Wrong Method. | |
| The National Medical Union. | |
| **The Institutional Worker** ... ... (Suppl.) | 1 |
| Spare-Time Economy. | |
| **Our Bureau of Information** ... ... ,, | 2 |
| Employment and Training. | |
| The Sick and in Need. | |
| **Institutional Needs** ... ... ... ,, | 3 |
| **The Book Market** ... ... .. ,, | 3 |

